# Beyond torture checklists: an exploratory study of the reliability and construct validity of the Torturing Environment Scale (TES)

**DOI:** 10.1186/s12889-021-10384-w

**Published:** 2021-02-17

**Authors:** Pau Pérez-Sales, Raquel González-Rubio, Blanca Mellor-Marsá, Gonzalo Martínez-Alés

**Affiliations:** 1 SiRa/GAC Center, Madrid, Spain; 2 Department of Psychiatry, Hospital La Paz, Madrid, Spain; 3 Instituto de Salud Carlos III, Madrid, Spain; 4 Hospital Clinico San Carlos, Madrid, Spain; 5 Psychiatry Department, Universidad Autónoma de Madrid, School of Medicine, Madrid, Spain; 6 Columbia University Mailman School of Public Health, New York, USA

**Keywords:** Torturing environment scale, Torture, Psychological Torture, Ill-treatment, Istanbul protocol, Torture methods, Gender, Spain

## Abstract

**Background:**

Torture methods have traditionally been quantified using checklists. However, checklists fail to capture accurately both the almost infinite range of available methods of torture and the victims’ subjective experience. The *Torturing Environment Scale* (TES) was designed as a multidimensional alternative that groups torture methods according to the specific human function under attack. This study aims to do an exploratory assessment of the internal consistency reliability and discriminatory validity of the TES as part of a construct validity assessment in a sample of Basque torture survivors.

**Methods:**

We applied the TES to a sample of 201 torture survivors from the Istanbul Protocol Project in the Basque Country Study (IPP-BC) to profile torturing environments in detention. To estimate the internal consistency reliability of the scale, categorical omega values were obtained for each subscale of the TES. To assess its discriminatory validity, the “known groups” method was used comparing mean scorings by gender, state security forces involved in the detention, and decade (the 1980s to the present) when the events took place.

**Results:**

Men reported more physical pain, while women reported more attacks on self-identity and sexual integrity. The TES also showed significant differences as regards the security forces involved in the detention: Civil Guard (a militarised police) used more manipulation of the environment, threats, fear, pain and extreme pain, as compared to national and regional corps. Finally, although patterns of torture remained mostly unchanged across decades, more recent detentions included more emphasis on psychological attacks: context manipulation, humiliation linked to sexual identity, and attacks to meaning and identity. For all subscales of the TES, categorical omega values ranged from 0.44 to 0.72.

**Conclusion:**

The TES may be a useful tool in profiling torturing environments. Its sensitivity to key contextual variables supports the discriminatory validity of the scale. While some of the subscales showed an acceptable degree of internal consistency, others require further analysis to improve reliability. The scale provides unique insights into the profile of contemporary torture. It will allow for future quantitative research on the relationship between different torturing environments and the medical and psychological consequences thereof.

## Background

### The challenge of measuring torture in research

Testimonies of torture victims across different cultures and ages have shown that the list of torture methods is as vast as the perpetrator’s imagination. This heterogeneity makes it challenging to conduct research in this field. Nonetheless, there have been many efforts to classify torture methods [1] and collect relevant information in a way that is useful for human rights research.

Traditionally, checklists have been the most common classification system. As a result, several notable scales have been developed over the last couple of decades including, to name a few, the Exposure to Torture Scale [2], the Allodi Torture Scale [3], and the Torture Checklist [4]. A recent review [5] collected up to 48 different questionnaires of war-related events (including torture), ranging in length from 8 to 164 violence-related items. In fact, most are not questionnaires but semi-structured interviews, designed to be used during rapid assessments in refugee camps, as an aid to clinical histories in rehabilitation centres, or in forensic evaluations for asylum claim processes. None of these checklists has been validated [5, 6], nor have their psychometric properties been published. However, they are useful as they provide a structured collection of data during therapy or as part of the documentation of torture.

Torture severity measurements are more refined versions of checklists. Half of the studies in Green et al.’s review [5] derived scores by merely summing the number of different types of abuse suffered (whether or not considered to be torture). A small number of studies also took into account the frequency and duration of the torturing techniques used. However, none of these measures includes the subjective perception of the impact of each torture method. Only the Semi-Structured Interview for Survivors of Torture [2] operationalises torture severity by calculating the total number of types of torture (from a list of 44 events), frequency of exposure to torture, duration of detention, and perceived severity of each type of experience of torture (i.e. distress) rated using a 5-point Likert scale. The Semi-Structured Interview for Survivors of Torture was designed to be used in the Balkans. Therefore, its applicability in other contexts, where torture methods change, remains unknown.

In summary, existing checklists provide a rough and inaccurate measure of torture that fails to capture both the full range of combinable torture methods available and the victims’ subjective experience.

### The torturing environment scale (TES)

The TES was designed as an alternative measure that groups torture methods according to *which human function is under attack* [7]. Hence, it depicts the profile of a given torture environment following a teleological approach, which means an alternative outlook measuring phenomena (torture methods) in terms of the purpose they serve (i.e. manipulation of environments, fear-producing actions, actions targeting identity, actions targeting the sense of belonging, actions targeting gender and sexual identity etc.) rather than how this is in practice done. Torture methods are as infinite as is the imagination of the perpetrator, and trying to list them could be an endless process. The TES addresses this problem by grouping them according to their purpose in the overall objective of breaking the self of the person. It is designed as a complement to the Istanbul Protocol, the United Nations standard for documentation of torture, and it can also be used as a monitoring tool for detention centres. It was derived from a three-layer identity-based theoretical model of torture formulated by the first author and described elsewhere [7] that puts in relation clusters of basic human needs (primary physiological functions, relation to the environment, need for safety, physical integrity, self, and identity) with different types of attacks inflicted, possible systems impacted (systems of conscious mind, fight and defence, secondary emotions, higher cognitive functions and ego, metacognitive functions), and consequences produced (brain, affect and anxiety circuits, and higher functions) (pp 258–270). The elaboration and structure of the TES as a corollary of the theoretical model has been extensively described in previous work [7]. In short, the TES includes 72 items distributed in four sections. The core section is Section I (Assessment of the Environment) that has 44 items distributed in 8 conceptual or teleological blocks (see Table 1). Values for each block range from 0 to 16. Additionally, it is complemented by Section II – Relational Pattern (10 items that describe the interaction between torturer and survivor), Section III on Legal Criteria according to the United Nations Convention against Torture (UNCAT) definition (6 items) and Section IV (12 items) on Medico-Psychological Findings suggestive of ill-treatment or torture. Finally, the Scale also includes a Physical Versus Psychological Torture (PPT) Index (scoring and interpretation available on request). The TES is available free online in English, Spanish and French. Researchers can introduce data and obtain their results in both graphic and Excel formats (www.psychosocial.info).

**Table 1:** Torturing Environment Scale. Section 1 - Conceptual Blocks and Items

Section 1 - Torturing Environment		
Block 1. Contextual manipulations	Attacks on essential body functions that allow staying oriented.	1.Inhuman conditions detention 2. Environmental manipulation 3. Basic physiological functions 4. Sleep dysregulation 5. Handling of time 6. Sensory deprivation 7. Mind-altering methods
Block 2. Fear-producing actions	Attacks to the need for security	9. Hopes and expectations 10. Threats to the person 11. Threats against family 12. Lack of information 13. Experiences of near-death 14. Witnessing others torture 15. Phobias
Block 3. Pain-producing actions	Attacks to the body - Mild to severe pain, prolonged in time.	17. Beatings 18. Battles against oneself 19. Exhaustion exercises
Block 4. Extreme pain – mutilation - death	Attacks to the integrity of the body – Excruciating pain – Permanent Damage - Death	21. Extreme pain 22. Mutilation 23. Brain damage
Block 5 – Sexual Integrity	Attacks to identity linked to gender	25. Humiliation 26. Sexual assault 27. Rape
Block 6 – Attachment and need to belong	Attacks to identity in relation	29. Solitary confinement 30. Breaking social bonds 31. Manipulation of affect
Block 7 – Actions targeting identity	Attacks to self	33. Beliefs and worldviews. 34. Helplessness induced 35. Instilling guilt 36. Induced shame 37. Induced humiliation 38. Violation of moral principles 39. Installing goals and identity
Block 8 – Coercive interrogation	Manipulation through dialogue and interrogation	41. Conditions 42. Style of interrogation 43. Deception/manipulation

#### Torturing environments

The TES shifts from summing up torture methods, to *measuring torturing environments*. We define a torturing environment as a milieu that creates the conditions for torture. It is built by contextual elements, conditions and practices that obliterate the will and control of the victim, exposing the self. In this sense, the environment will be considered as leading to cruel, inhuman or degrading treatment (CIDT) or torture when it has been generated intentionally for any of the purposes stated in the United Nations definition. The creation of a Torturing Environment can include one or more of the following: a) attacks on primary needs and relation to the environment; b) attacks on the need for safety and physical integrity, including pain, threats and fear; and c) attacks to the self and identity, including individual, group and collective dimensions of identity.

### Torture in the Basque country

According to official records and non-governmental organisations (NGOs), torture has been part of the Basque country reality as part of the policies of social control under Franco’s dictatorship and remained during democracy as part of anti-terrorism policies against separatist groups. According to official figures provided by the Basque Government in the framework of the peace and reconciliation process [8], 4113 persons were subjected to torture between 1960 and 2015 [9]. However, data collection has not been completed, and the number is likely to increase with time. Between the years 2012 and 2015, *The Istanbul Protocol Project in the Basque Country* (IPP-BC) *Working Group* conducted a study using a protocol of enhanced credibility assessment with an initial sample of 45 people [10], subsequently enlarged to 200 individuals who underwent incommunicado detention between the years 1965 and 2015. In incommunicado detention, the detainee is denied access to family members, an attorney, or an independent physician. The only contact allowed is with the interrogators. Incommunicado detainees may be held and interrogated by three security forces: The *Civil Guard,* a public security corps with military status and national scope that is part of the State Security Forces, the *National Police,* an armed institution of a non-military nature depending on the Ministry of Internal Affairs, and the *Ertzaintza*, a similarly non-military local armed force depending on the Autonomous Government of the Basque Country. In the experience of the IPP-BC Working Group members and according to human rights reports and qualitative data [11–15], the detainee’s gender, the type of security forces involved and the decade in which the person was tortured were the most relevant variables shaping the experience of torture survivors in the Basque country.

#### Aim of the study

This research aims to conduct an exploratory reliability and construct validity assessment of the TES in a sample of Basque torture survivors. The objectives are to estimate the internal consistency reliability of the TES and to assess its capacity to discriminate between groups that are expected to have different values in the TES (discriminative validity). The study was conducted to test three hypotheses based on the results of the initial 45 IP Pilot Study [10]: (a) Men and women have different profiles of torturing environments, with men experiencing more physical and pain-based torture and women experiencing more psychological manipulation and sexual torture. (b) Militarised police are associated with a harsher and more physical torturing environment than non-militarised police forces. (c) Earlier cohorts report more physical and pain-based torture methods, as compared to more psychological torture experienced/reported in later ones.

## Methods

### Design, participants and procedure

A cross-sectional study was conducted using a random sample of torture survivors from the official records of the Basque Government between 1960 and 2012 [15]. Data was collected between October 2012 and September 2013. We calculated a sample size of 200 in order to detect 2-point differences in the scale between individuals detained before or after the year 2000, considering a 90% power and a 2-sided 0.05 alpha level, assuming SD = 4 from the general description of data.

The experts (one psychiatrist, 16 clinical psychologists, three medical doctors; *n* = 20) underwent training in the use of the scale from the first author, including theoretical explanations and simulation exercises using video recordings of actual cases. Inter-rater reliability was substantial to high - Kappa coefficient of agreement between experts was 0.89 [16].

Each case was initially evaluated by one of the mental health forensic experts (psychiatrist or clinical psychologist). After obtaining informed consent, they conducted extensive clinical interviews using a semi-structured format based on the Istanbul Protocol. The interviews included a battery of tests and were recorded on tape or video and transcribed. The medical expert undertook an independent assessment. Both then jointly completed the *Torturing Environment Scale*.

### Statistical analysis

We performed bivariate analyses using non-parametric (Kruskal-Wallis and Mann-Whitney U) tests including (a) conceptual blocks, (b) individual items and (c) PPT Index. Standardised effect size estimates r and epsilon-squared were calculated for group differences in conceptual blocks scores. As measures of internal consistency, we obtained categorical omegas (ω_c_) as well as their related confidence intervals (CI) for each Block of Section I and Section II. Categorical Omega is a more appropriate index of internal consistency than other alternatives such as Cronbach’s alpha [17, 18]. All descriptive analyses were carried out using SPSS (Statistical Package for Social Sciences v22), and all categorical omegas and confidence intervals were calculated using R, MBESS package. We set the level of significance at *p* < 0.05, and Bonferroni correction was used when analysing differences between categories of independent variables.

## Results

### Characteristics of participants

The sample was comprised of 201 survivors of torture (SOT) (Table 2). One-hundred fifty-nine (79.1%) were men, and 42 (20.9%) were women. The average age at detention was 24 (IQR = 22–30). Time elapsed between detention and medical interview varied from 2 to 40 years, with 20.3 years on average. Most of them had been detained by either the Civil Guard (38.3%) or the National Police (30.8%). It is of note that 43 subjects (21.4%) had been detained more than one time by different security bodies. In such cases, we asked the interviewee to report considering only the worst detention in his/her view. In a preliminary analysis, we compared both subsets and found no differences in any of the scales of the TES.

**Table 2:** Characteristics of the sample and conditions of detention

**Sex** n (%)	Men	159 (79.1)
	Women	42 (20.9)
**Age** median (IQR)		54 (39–60)
**Level of education** n (%)	Primary	34 (16.9)
	Secondary	102 (50.7)
	Tertiary	65 (32.3)
**Age at detention** median (IQR)		24 (22.3)
**Year of detention** n (%)	Before 1979	34 (16.9)
	1980–1989	71 (35.3)
	1990–1999	33 (16.4)
	After 2000	63 (31.3)
**Security forces involved in the detention** n (%)	Civil Guard	77 (38.3)
	National Police	62 (30.8)
	Ertzaintza (Basque Police)	19 (9.5)
	More than one detention	43 (21.4)
**After interrogation** n (%)	Freedom without charge	71 (35.3)
	Freedom with charge	13 (6.5)
	Pre-trial detention	117 (58.2)
**Days in incommunicado detention** median (IQR)		4 (3–6)
**Total** n (%)		201 (100)

*IQR* Interquartile range

One-hundred seventeen individuals (58.2%) were sent by a judge to prison pending trial (pre-trial detention), in most of the cases based on self-incriminating statements signed during torture without applying the exclusionary rule. The rule establishes, under Article 15 of the UN Convention against Torture, there is an absolute prohibition to invoke as evidence against a person any information obtained using cruel, inhuman or degrading treatment or torture.

### Torturing environment as measured by the TES

**Fig. 1:**
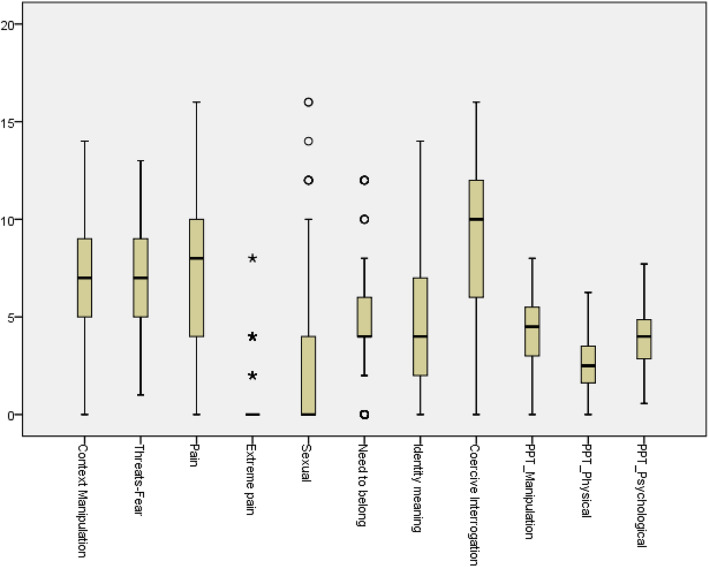
Distribution of the scores of Torturing Environment Blocks (*N* = 201). PPT = Physical versus Psychological Torture Index

**Fig. 2:**
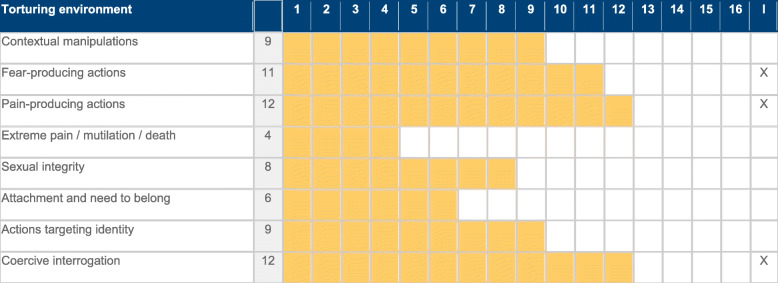
Output of the software program. 1 to 16 shows the pre-eminence of this profile of torturing methods in each of the 8 conceptual blocks. Column I indicates the blocks that are considered by victims as the ones that most affected them

Figure 1 plots the median, interquartile range (IQR) and maximum and minimum values for each of the eight blocks. Figure 2 shows the average score for each block, as shown by the software output. Both figures indicate that torture in the Basque country targeted all aspects of a human being. However, the overall pattern was based on a combination of manipulation of the environment (conditions of cells, hooding, manipulation of time, hunger, etc.), threats (more torture, relatives), pain-producing actions (stress positions, slaps, punches), coercive interrogation (emotional and cognitive manipulation, deceiving), and attacks against personal identity. Use of sexual torture was present though limited, and very few cases of extreme and excruciating pain were reported.

It is important to note that, from a conceptual point of view, the intensity of the attack reflected in the output does not necessarily correlate with the suffering of the victim. The scale measures the acts of torture perpetrated and the subjective experience of the victim, but this does not mean that the suffering of victims can be compared. Any implication in terms of comparing more or less severity of suffering in the experiences of torture survivors would be erroneous and beyond the scope of the scale.

### Discriminatory validity

#### Between-groups analysis

Tables 3, 4, 5 and 6 show the differences by blocks on the three key variables.

**Table 3:** Torturing Environment Blocks by Gender (*N* = 201)

Blocks median (IQR)	Men (*N* = 159)	Women (*N* = 42)	Standardised Test Statistic	Effect Size Estimate (r_ab_)
Context Manipulation	7 (5–9)	7.5 (4–10)	0.463	0.033
Threats-Fear	7 (5–9)	7.5 (5–10)	0.604	0.043
Pain	8 (4–10)	6 (2–8)	−2.798**	0.197
Extreme Pain	0 (0–0)	0 (0–0)	−1.052	0.074
Sexual integrity	0 (0–4)	3 (0–8)	2.261*	0.159
Need to belong	4 (4–6)	4 (4–4)	0,006	0.001
Identity, Meaning	4 (2–6)	6 (4–8)	2.423*	0.171
Coercive Interrogation	10 (8–12)	9 (4–12)	−0.734	0.052
PPT-Manipulation	4.5 (3.5–5.5)	4.75 (3–6)	0.033	0.002
PPT-Physical	2.6 (1.7–3.7)	2.2 (0.5–3)	−2.539*	0.179
PPT-Psychological	4 (3–4.6)	4.1 (2.6–5.6)	0.759	0.054

*p-value < 0.05, **p < 0.01

**Table 4:** Gender analysis of torture methods. Summary of significant results

Male	Female
Dry asphyxia (“bolsa”)** Wet asphyxia (“Submarino”)* Blunt trauma (punches, kicks, slaps)* Exhaustion exercises (push-ups, etc.)** Harsh environment of interrogation*	Sensory deprivation / blindfolding* Unsurmountable fears / phobias* Humiliation related to sexual identity (i.e. forced nakedness)** Manipulation of affect*

*p < 0.05, ** *p* < 0.01

**Table 5:** Torturing Environment Blocks by security forces involved in detention (*n* = 158)

Blocks median (IQR)	Civil Guard	National Police	Ertzaintza	Test statistic	Epsilon-squared estimate
Context Manipulation	8 (5–10)^a^	6 (4–8)^a^	7 (5–9)	7.994*	0.051
Threats-Fear	8 (5–10) ^a, b^	6 (5–8) ^a^	5 (3–8) ^b^	9.789**	0.062
Pain	8 (6–12) ^a^	6 (4–8)	4 (2–6) ^a^	10.797**	0.069
Extreme Pain	0 (0–3) ^a, b^	0 (0–0) ^a^	0 (0–0) ^b^	13.676**	0.087
Sexual integrity	2 (0–8)	0 (0–4)	0 (0–4)	5.518	0.035
Need to belong	4 (4–6)	4 (4–4)	4 (4–4)	1.081	0.007
Identity, Meaning	4 (3–7)	5 (3–7)	4 (2–7)	0.703	0.004
Coercive Interrogation	10 (6–12)	10 (6–12)	10 (8–12)	1.820	0.012
PPT-Manipulation	5 (3.5–6) ^a^	4 (3–5) ^a^	4.5 (3–5.5)	8.146*	0.052
PPT-Physical	3 (2–4.5) ^a, b^	2.3 (1.8–3) ^a^	15 (0.5–2.25)^b^	18.743**	0.119
PPT-Psychological	4 (2.9–5.1)	4 (2.6–4.6)	3.9 (2.4–4.4)	2.036	0.013

The superscript letters are the same in those groups between which there are differences. In the Threats-Fear variable, the differences between groups have a p-value at the limit of statistical significance. *p-value< 0.05, **p < 0.01

**Table 6:** Torturing Environment Blocks by decade of detention (N = 201)

Blocks median (IQR)	Before 1979	1980’	1990’	2000–2010	Test statistic	Epsilon-squared estimate
Context Manipulation	6 (4–9)	6 (4–8)^a^	7 (4–9)	8 (6–9)^a^	8.388*	0.053
Threats-Fear	6.5 (5–10)	7 (5–9)	7 (4–10)	7 (5–8)	0.288	0.002
Pain	8 (4–10)	8 (6–10)	6 (4–10)	8 (4–10)	1.934	0.012
Extreme Pain	0 (0–4)	0 (0–0)	0 (0–0)	0 (0–0)	7.144	0.046
Sexual integrity	0 (0–2) ^a^	0 (0–4)	2 (0–4)	2 (0–8) ^a^	9.757*	0.062
Need to belong	4 (4–4)	4 (0–6)	4 (4–4)	4 (4–6)	4.182	0.027
Identity, Meaning	4 (2–5) ^a^	4 (2–6) ^b^	4 (2–6)	6 (4–8) ^a, b^	14.158**	0.090
Coercive Interrogation	9 (4–12)	8 (6–10)	10 (6–12)	10 (8–12)	7.390	0.047
PPT-Manipulation	4 (3–5.5)	4 (3–5.5)	4.5 (3.5–5.5)	5 (4–6)	8.204*	0.052
PPT-Physical	2.5 (2–4.5)	2,6 (2–3.5)	2.3 (1.5–3.8)	2,5 (1–3.5)	2.064	0.013
PPT-Psychological	3.7 (2.3–4.3)^a^	3.8 (2.9–4.6)^b^	3.9 (2.9–4.8)	4.3 (3.3–5.4) ^a, b^	10.208*	0.065

The superscript letters are the same in those groups between which there are differences. In the Context Manipulation variable, the differences between groups have a *p*-value at the limit of statistical significance

*p-values< 0.05, **p < 0.01

#### Gender

More physical pain was used on men; with women, torturers were more likely to use attacks on identity (gender, family, political involvement). According to the PPT index, men reported suffering more physical than psychological methods.

The analysis item by item (available on request) is summarised below (Table 4).

#### Security forces involved in detention

The interrogations by the Guardia Civil were harsher than those of other security forces (Table 5). They were more likely to use manipulation of the environment (conditions of the cell, hooding, blindfolding), threats and fear (including mock executions and dry and wet asphyxia), moderate pain (blunt trauma: punches, kicks, slaps, etc.; exhaustion exercises: push-ups, etc.), and extreme pain (positional torture [“quirófano”] and electric torture).

#### Decade of detention

There were small differences concerning the decade of detention (Table 6). From the 1980s to the last cases in 2012, torture methods mostly remained unchanged. However, there was a tendency over time and especially in the later years towards more context manipulation (blindfolding and hooding), humiliation linked to sexual identity (harassment and deprecation), and attacks to meaning and identity. More recent interrogations were more likely to use more emotional and cognitive manipulation. In general terms, and according to PPT index, Torture slightly evolved from Physical to Environmental and Psychological.

### Internal consistency/reliability

Categorical omega and confidence intervals for each block were: Contextual manipulations ω_c_ = 0.63 (0.43–0.71); Fear ω_c_ = 0.44 (0.10–0.56); Physical pain ω_c_ = 0.53 (0.39–0.62); Extreme pain (not enough cases), Sexual integrity ω_c_ = 0.71 (0.52–0.79); Need to belong, acceptance and care ω_c_ = 0.48 (0.35–0.59); Identity, control, meaning and purpose ω_c_ = 0.72 (0.60–0.78); and Coercive Interrogation Techniques ω_c_ = 0.71 (0.61–0.78). Categorical omega for Manner of Interaction was ω_c_ = 0.53 (1.98–76.61).

## Discussion

The concept of Torturing Environments is a new one that shows promise in the assessment of problematic conditions in which the combination or accumulation of coercive methods might amount to torture [7], as the Special Rapporteur against Torture has recently acknowledged [19]. The Torturing Environment Scale (TES) is the first tool to assess the construct systematically. The present study is the first to apply the TES to a representative sample of torture survivors and assess its psychometric characteristics. According to the “known group” methods [20], the TES showed differences by critical variables in the analysis of the experience of SOTs and exhibited agreement in the expected direction as suggested by previous studies, qualitative reports, and field workers and human rights groups from the Basque Country [8, 13, 15, 21–25]. Moreover, our data suggest that the TES has good construct validity as measured by its good discriminative power. In addition, our results are highly consistent with previously published data from the same sample that explored torture methods and long-term sequelae applying the Istanbul Protocol checklist of torture methods [12, 22, 26].

Our results support our initial hypothesis of a different profile of torture by gender. Accordingly, the TES was able to discriminate gender profiles in torturing environments in the Basque country, as described in previous studies in this context [12], and in line with data from other contexts like Balkans [27], French-Algerian War [28] and South Africa [26], among others. We also found differences in the torturing profile between the different security forces involved in the detentions. The Civil Guard used more manipulation of environment, fear, pain, and attacks to identity as compared to National Police and local Basque police (Ertzaintza). These results are in accordance with previously published qualitative research conducted on similar samples, where Civil Guard was described as using more violent methods such as electrodes and bathtub torture whereas survivors more often described National Police and Ertzaintza as conducting interrogations with blows and very long waits but without harsh physical assaults [7]. Finally, our third hypothesis regarding the evolution of torture environments over time was only partially supported. Although the use of psychological methods of torture, aimed to attack the sense of identity and self, has increased through the years, physical pain as a fundamental approach has remained throughout the decades. In qualitative studies, survivors from the Basque country reported that torture evolved with time to more psychological methods and that torture was mostly physical during the 1980s [13]. However, this does not appear to be confirmed by our data, most likely due to the small number of participants in our study who were tortured during the 1980s. We cannot discard also recall problems. We have not found directly comparable studies from other countries, although a sample of torture survivors from the Yugoslavian civil war reported gender differences in torture methods and sequelae that coincide with our results [27]. Taken all together, there is substantial evidence that there is a tendency over time and especially in the later years to target psychological processes in an attempt to leave no marks and have shorter detention periods. However, interrogators do not renounce physical coercion. Further studies will have to explore if this was specific to the Basque context or if it is part of a more global tendency.

The TES showed different degrees of internal consistency across its eight Conceptual Blocks. Values of categorical omega were acceptable for Sexual Integrity, Identity and Coercive Interrogation but very low for Fear and Need to belong. Different factors may have influenced these low values. Some factors are related to the structure of the scale, as suggested by low intercorrelations among individual items or a small number of items in some blocks. A potential workaround would be to increase the number of items in the blocks with lower omega values [29]. Also, the sample might be too homogeneous in terms of the experience of detention and interrogation [30]. For instance, the conceptual block Need to belong includes only four items, with most participants scoring positive as all detainees were submitted to incommunicado detention. The homogeneity of the sample is, at the same time, the principal strength and one of the limitations of the study. Additional studies are needed in other more heterogeneous samples. The information arising from the analyses within this group is especially useful while conceptualising the Basque Country conflict. More exploratory studies conducted in other contexts and conditions will add essential knowledge regarding the generalizability of the results and the usefulness of the scale. However, the TES has confirmed what was suggested by previous qualitative studies based on the Istanbul Protocol, the standard of reference in the assessment of torture allegations, which suggests the robustness of the scale [26]. Finally, there is also the concern that some subjects, especially those detained during the 1980s, and those detained by Ertzaintza, were underrepresented.

We consider this study to have several strengths. First, it shows results from an extensive sample of torture survivors assessed using the Istanbul Protocol and the TES. The reliability of these expert forensic assessments has been appraised elsewhere [26]. The magnitude of the sample and the careful process of applying the TES with a high inter-rater k coefficient suggests that it can be used in research without intensive training. The scale is offered free to the scientific community to use through a website of the Project (www.psychosocial.info).

The TES is not a substitute for a clinical or an ethnographic interview. Instead, it helps to organise the information using a teleological approach. By *teleological*, we mean that the focus of the TES is not on listing methods, but the target of each method in terms of a human mind-body system. Hence, it opens new avenues for quantitative research on contemporary torturing environments and its correlation with clinical variables. The TES is by no means a measure of the suffering of persons and should not be used with that purpose. It also offers potential for monitoring visits to institutions and in the framework of public health studies.

### Limitations

There are some limitations of the study. (a) The sample is very homogeneous, and this might influence the validation of those subscales with lower scores. (b) It includes torture survivors from 40 years. In some instances, time might distort the perception of trauma (c) People tortured in the 80s and people tortured by Ertzaintzz might be underrepresented in the sample.

## Conclusions

The TES can be a useful tool in detecting profiles of torturing environments. Here it is shown to be sensitive to key contextual variables, supporting the discriminative validity of the scale. While some of the subscales demonstrated an acceptable degree of internal consistency, others require further analysis to improve reliability. The TES provides unique insights into the way that the torturing process is thought about and can help understand the physical and psychological impacts of torture on survivors and its clinical implications. Future studies should explore further the relationship between specific profiles of torturing environments and clinical impacts and in particular, those methods that should require special international regulation.

## Data Availability

The datasets used and analysed during the current study are available from the corresponding author on reasonable request.

## Abbreviations

TES: Torturing Environment Scale
IP: Istanbul Protocol
IPP-BC Working Group: The Istanbul Protocol Project in the Basque Country Working Group
PPT Index: Physical versus Psychological Torture Index
UNCAT: United Nations Committee Against Torture
SOT: Survivors of Torture
AVPD: Basque Agency for Data Protection
UPV/EHU: University of the Basque Country
